# The Learning Curve and Inter-Observer Variability in Contouring the Hippocampus under the Hippocampal Sparing Guidelines of Radiation Therapy Oncology Group 0933

**DOI:** 10.3390/curroncol29040210

**Published:** 2022-04-08

**Authors:** Monika Konopka-Filippow, Ewa Sierko, Dominika Hempel, Rafał Maksim, Natalia Samołyk-Kogaczewska, Tomasz Filipowski, Ewa Rożkowska, Stefan Jelski, Beata Kasprowicz, Eryka Karbowska, Krzysztof Szymański, Kamil Szczecina

**Affiliations:** 1 Department of Oncology, Medical University of Bialystok, 15-089 Białystok, Poland; mkonopka@onkologia.bialystok.pl (M.K.-F.); domhem@wp.pl (D.H.); 2 Department of Radiotherapy I, Maria Sklodowska-Curie Bialystok Oncology Centre, 15-027 Białystok, Poland; rafalmaksimi@gmail.com (R.M.); natalia.samolyk@gmail.com (N.S.-K.); tfilipowski@onkologia.bialystok.pl (T.F.); erozkowska@onkologia.bialystok.pl (E.R.); 3 Department of Radiology, Maria Sklodowska-Curie Bialystok Oncology Centre, 15-027 Białystok, Poland; sjelski@onkologia.bialystok.pl (S.J.); jbk35@wp.pl (B.K.); ekarbowska@onkologia.bialystok.pl (E.K.); 4 Department of Physics, University of Bialystok, 15-245 Białystok, Poland; k.szymanski@uwb.edu.pl (K.S.); kszczecina@onkologia.bialystok.pl (K.S.)

**Keywords:** hippocampal sparing, hippocampal avoidance, brain radiotherapy, RTOG delineation atlas, contouring, learning curve

## Abstract

Hippocampal-sparing brain radiotherapy (HS-BRT) in cancer patients results in preservation of neurocognitive function after brain RT which can contribute to patients’ quality of life (QoL). The crucial element in HS-BRT treatment planning is appropriate contouring of the hippocampus. Ten doctors delineated the left and right hippocampus (LH and RH, respectively) on 10 patients’ virtual axial images of brain CT fused with T1-enhanced MRI (1 mm) according to the RTOG 0933 atlas recommendations. Variations in the spatial localization of the structure were described in three directions: right–left (X), cranio-caudal (Y), and forward–backward (Z). Discrepancies concerned three-dimensional localization, shape, volume and size of the hippocampus. The largest differences were observed in the first three delineated cases which were characterized by larger hippocampal volumes than the remaining seven cases. The volumes of LH of more than half of hippocampus contours were marginally bigger than those of RH. Most differences in delineation of the hippocampus were observed in the area of the posterior horn of the lateral ventricle. Conversely, a large number of hippocampal contours overlapped near the brainstem and the anterior horn of the lateral ventricle. The most problematic area of hippocampal contouring is the posterior horn of the lateral ventricle. Training in the manual contouring of the hippocampus during HS-BRT treatment planning under the supervision of experienced radiation oncologists is necessary to achieve optimal outcomes. This would result in superior outcomes of HS-BRT treatment and improvement in QoL of patients compared to without HS-BRT procedure. Correct delineation of the hippocampus is problematic. This study demonstrates difficulties in HS-BRT treatment planning and highlights critical points during hippocampus delineation.

## 1. Introduction

Brain irradiation (BI) is an essential treatment modality in a variety of oncological disorders and includes whole brain radiotherapy (WBRT) or stereotactic radiotherapy (SRT) for brain metastases (BM), prophylactic cranial irradiation (PCI) for small cell lung cancer patients, cranial irradiation or SRT in primary brain tumors (PBT) and other cerebral pathologies [1,2,3]. Despite many benefits of cranial irradiation, e.g., improved local control (LC) and overall survival (OS) [4,5], focus has recently shifted to the adverse events associated with the treatment, particularly neurocognitive decline and a decreased quality of life (QoL) in irradiated patients [6,7]. Clinical evidence indicates that three months after BI, patients suffer from impairment in memory function, in particular immediate and delayed recall [8], which is the result of radiation-induced injury to the brain neurogenic zones including the hippocampus [4]. Despite the technical aspects of hippocampal avoidance during BI, considerable controversy surrounds proper assignment of patients to the procedure without reducing the benefits of RT in a given clinical situation. Evidence has been accumulating in recent years that hippocampal avoidance (HA) should be followed in cancer patients with four or more BM assigned to WBRT or in patients diagnosed with small cell lung cancer assigned to PCI [9,10].

The hippocampus is a paired brain structure, situated in the ventromedial section of the temporal lobe, located laterally to the temporal horn of the lateral ventricle. The bodies of the hippocampi consist of the dentate gyrus and the cornu ammonis areas, and their parts of the limbic system [6]. The hippocampus plays an essential role in learning, spatial processing, memory formation, retrieval and consolidation of information [11]. The hippocampus contains active neural stem cells (NSCs) in two “niches”: the subgranular zone of the dentate gyrus (SGZ)—a crucial center for learning and memory function—and the subventricular zone (SVZ) [12]. NSCs are very sensitive to ionizing radiation which causes their apoptosis (reaching a peak as early as 12 h after irradiation) followed by a decrease in the number of proliferating cells in SGZ [6,13]. Finally, bilateral or unilateral radiation-induced injury to NSC niches results in deterioration of neurocognitive function [14]. Initial reports from the first prospective phase II Radiation Therapy Oncology Group (RTOG) 0933 study suggest that avoiding irradiation of the hippocampus in patients with BM reduces the risk of cognitive decline [15].

Modern radiotherapy (RT) techniques such as tomotherapy, intensity modulated radiation therapy (IMRT) or volumetric modulated arc therapy (VMAT) open the possibility to selectively spare desired structures. However, proper contouring of SGZ in the hippocampus is a prerequisite for sparing this structure from excessive irradiation [9,16].

Target volume and organ at risk (OAR) contouring is one of the most important steps in RT treatment planning process. However, it is a difficult undertaking since the hippocampus, in contrast to other structures in the brain such as the brainstem or optic chiasm, is embedded within the temporal lobe. Preliminary online recommendations for hippocampus delineation have recently been published by RTOG (RTOG 0933 Hippocampal Sparing Atlas) [17]. The recommendations confirmed previously published proposals that the dose of tolerance which preserves normal neurocognitive function in patients after BI is 16 Gy in conventionally fractionated RT [13]. Delineation of SGZ only, which contains a large population of NSCs, instead of the entire hippocampus, has also been suggested [18]. According to RTOG recommendations, the hippocampus should be contoured on the brain axial images of computed tomography (CT) fused with magnetic resonance (MRI) axial FLAIR, axial T2-weighted and gadolinium contrast-enhanced T1-weighted sequence acquisitions with the preferred slice thickness of at least 1.25 mm [19]. Delineation of the hippocampus starts with the most caudal extent of the crescent-shaped floor of the temporal horn of the lateral ventricle and continues through the hypointense grey matter located medially to the cerebrospinal fluid (CSF) hypointensity, inside of the temporal horn. On the coronal axis, the hippocampus is located medially to the ventricles. On the sagittal and coronal axes, separation between the tail of the hippocampus and the crus of the fornix can be observed [19].

### Aim of Study

The aim of the study was to assess the learning curve and potential inter-observer variability of manual hippocampus delineation during hippocampal-sparing brain radiotherapy treatment planning.

## 2. Material and Methods

The assessment of the quality of hippocampal contouring was performed in the Radiotherapy Department of Maria Sklodowska-Curie Bialystok Oncology Centre, Bialystok, Poland. The study was approved by the Bioethics Committee of the Medical University of Bialystok, Poland (consent No R-I-002/210/2016, date 15 October 2015). Prior to the introduction of the hippocampal-sparing procedure into everyday clinical practice, 10 doctors (7 radiation oncologists and 3 radiologists) delineated the left and right hippocampus (LH and RH, respectively) in the area of the hippocampal dentate gyrus on 10 patients’ virtual axial images of brain CT, fused with T1-weighted MRI without contrast enhancement (performed every 1 mm), according to the RTOG 0933 atlas recommendations [19]. All participating doctors delineated LH first, followed by RH. In all cases, hippocampus delineation was an individual learning process. None of the participating doctors had contoured the hippocampus previously. The status of hippocampus delineation was not discussed with the participants during the study. Eight doctors were right-handed, while two were left-handed. Four radiation oncologists had more than 25 years of experience in contouring brain structures, three had more than three years of experience, while three radiologists had at least 10 years’ experience in the diagnostic imaging of the brain. All doctors underwent training in hippocampus contouring prior to study commencement. A total of 10 patients (4 female, 6 male) with no history of neurological, psychiatric, or cognitive impairment disorders, diagnosed with PBT or BM in RPA prognostic class I and II (Karnofsky performance score more than 70%) participated in this study. Mean patient’s age was 55.3 (range 37–68) years. Four patients diagnosed with non-small cell lung cancer, breast cancer, colon cancer had one BM, two patients diagnosed with non-small cell lung cancer, melanoma malignum had two BM and others with PBT (two with meningiomas and the rest with glioblastoma). The metastases, PBT or peritumor oedema were not located in temporal lobes.

**Figure 1:**
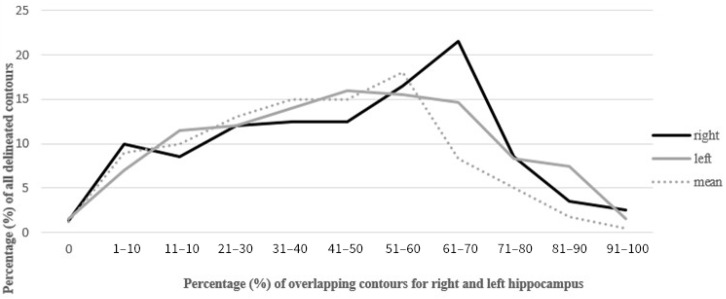
Percentage of overlap of delineated contours of the left and right hippocampus.

Visually determined hippocampus was manually delineated using elliptical ROIs (regions of interest) on consecutive slices (Figure 1). The hippocampal bodies were contoured as two separate structures without conjunction with the amygdala in all cases. Variations in the spatial localization of the hippocampi delineated by different doctors were described in three directions: right–left (X), cranio-caudal (Y), and forward–backward (Z), in relation to the reference contour of the hippocampus delineated by the most experienced radiation oncologist with expertise in neuroimaging, according to RTOG atlas recommendations [20]. It needs to be emphasized that previously delineated hippocampi were invisible to subsequently participants.

The physical algorithm was prepared by medical physicists for comparison of deviations in hippocampal contours in three-dimensional space. Variations in hippocampal contouring were assessed by comparing parameters (size, volume, location of the hippocampus, percentage of contour overlap) calculated from the physicians’ contours. There are no specific methods of the quantitative determination of 3D shape similarities. An appropriate measure of differences between investigated parameters has to be specified in each study. We selected a parameter called Hausdorf distance (HD), which describes deviations between delineated contours based on 3D spatial localization [19,21]. Comparisons were made using the paired samples’ *t*-test. Differences in the spatial localization as above of LH and RH separately of all hippocampi were statistically correlated using the corresponding hippocampal volumes and hippocampal T1-weighted MRI values, both independently and in combination. Reproducibility between the hippocampi contoured by different doctors in the same case was assessed using R vector, which measures a shift of the hippocampus in relation to the reference contour in all delineated cases.

Agreement between contoured hippocampi was assessed by degree of overlap using as well as the Dice coefficient, which ranges from 0 (no overlap) to 1 (complete overlap). The closer the result to 1, the greater the comparability of the two volumes in terms of size and location of contoured hippocampi.

For two separate volumes, the Dice coefficient was defined by:

Dice = 2 × (Volume 1 ‘overlap’ Volume 2)/Volume 1 + Volume 2

In study, the Dice coefficient was assessed for each delineated case of hippocampus compared to the reference contour of the hippocampi.

## 3. Results

### 3.1. Size and Volume of the Hippocampus

The mean volume of LH and RH (delineated dentate gyrus) was 1.9 cm^3^ and 1.8 cm^3^, respectively. The average sizes of the hippocampus were 1.7 cm and 0.9 cm in the Z and X axes, respectively. The largest differences were observed in the first three contoured cases: Z-axis deviation exceeded 5 mm in more than half of hippocampal contours. Hippocampal volumes in the first three delineated cases were larger (by more than 2 cm^3^) than in the subsequent seven cases.

### 3.2. Overlapping Contours

**Figure 2:**
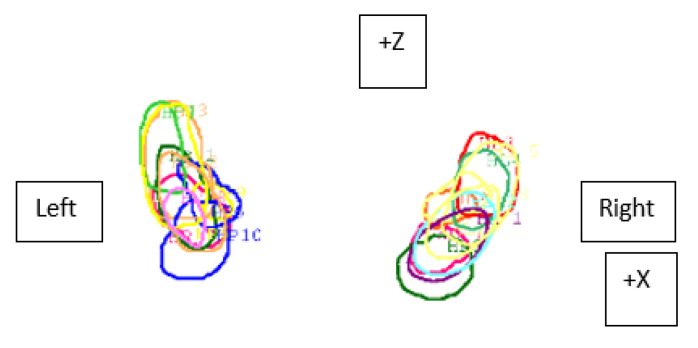
Visualization of contours of the left and right hippocampus delineated by 10 doctors and differences in their overlap (Z—forward–backward axis, X—right–left axis) in the sixth patient.

**Figure 3:**
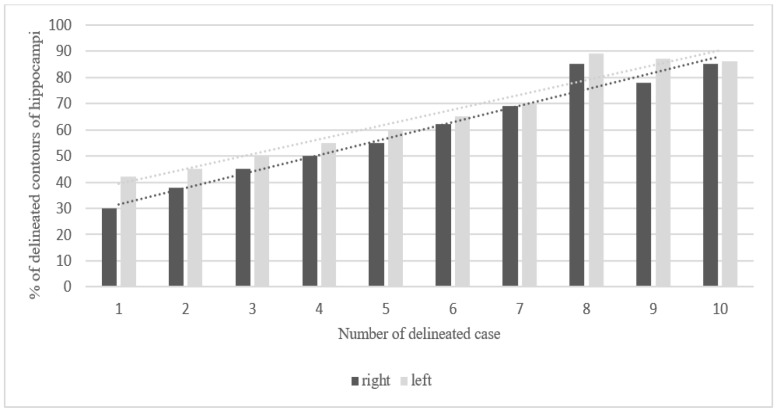
Steadily increasing learning curve in delineation of hippocampi. Percentage of overlapping contours of the right and left hippocampus in 10 consecutive cases (20 hippocampi were delineated).

The mean overlap of the hippocampal contour, which describes which part of a contour delineated by one doctor is included in the contours delineated by other doctors, ranged from 50% to 70%, whereas 20 out of all delineated contours did not overlap at all (in the first four delineated cases) (Figure 1, Figure 2 and Figure 3). A larger range of overlapping contours was observed for LH delineation.

### 3.3. Hausdorf Distance and Dice Coefficient

**Figure 4:**
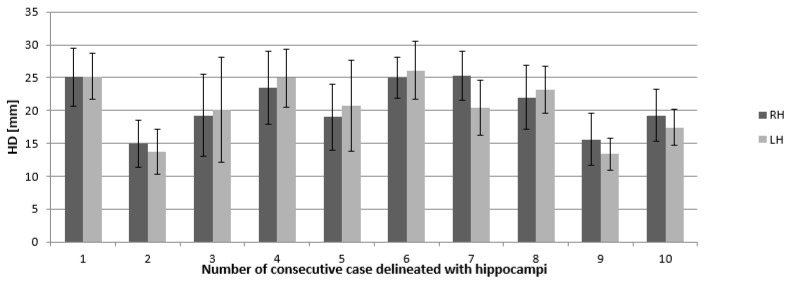
Mean Hausdorf’s distance (given in millimeters) and standard deviation indicating the 3D differences in right and left hippocampus delineation. Abbreviations: RH—right hippocampus, LH —left hippocampus, HD—Hausdorf distance.

**Figure 5:**
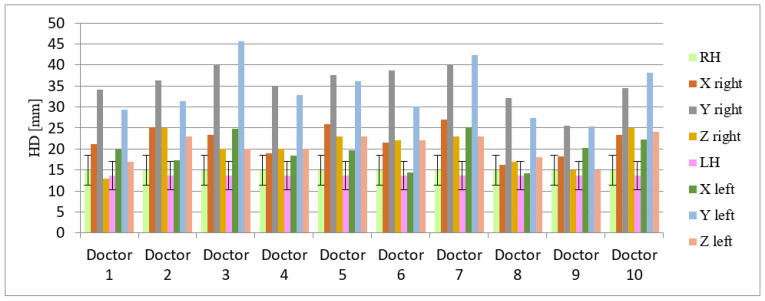
Hausdorf distance (in millimeters) indicating 3D variability in delineation of the left and right hippocampus by 10 different doctors (data presented for three axes: X, Y, Z). Abbreviations: HD—Hausdorf distance, LH—left hippocampus, RH—right hippocampus, axis: X—right–left, Y—cranio-caudal, Z—forward–backward. The doctors designated as ‘Doctor 1, 2, 8 and 9′ have more than 25 years of experience in radiation oncology in compared to ‘Doctors designated as 3, 7 and 10′ with more than 3 years of experience in radiation oncology. The radiologists were designated under the numbers 4, 5, 6.

Calculated HD illustrates averaged deviations between all contours in the RH and LH in all cases and they range from 1.3 to 2.6 cm (standard deviation (SD) for RH and LH were 4.43 and 4.47 cm, respectively). These values of HD are similar to each other in all cases (Figure 4 and Figure 5).

Whereas the average Dice coefficient calculated for hippocampi was 0.45 and 0.46 for left and right, respectively.

### 3.4. Discrepancies in Axis and Location

**Figure 6:**
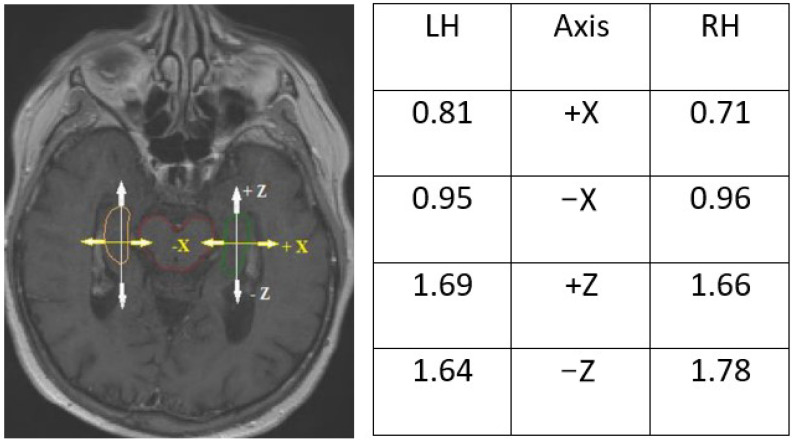
Deviations in hippocampal contours in the X- and Z-axes on the middle of the center of the brain stem visualized on T1-enhanced MRI scan. Symbols +/− indicate direction of deviation: +X towards outside of the brain stem, −X towards the brain stem, +Z forward, −Z backward, LH—left hippocampus, RH—right hippocampus.

**Figure 7:**
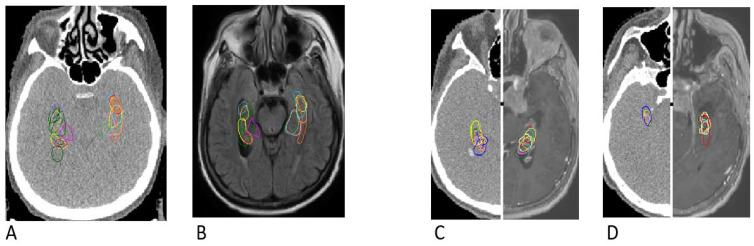
The largest discrepancies in the 1st case of hippocampus contouring (**A**,**B**) and the smallest discrepancies in the 9th case of hippocampus contouring (**C**,**D**) indicating a positive learning experience by doctors. Differences are visualized on computed tomography images (A, left C and left D) as well as on T1-enhanced MRI sequences (B, right C and right D).

The biggest differences in hippocampus delineation were observed in the area of the posterior horn of the lateral ventricle and near the quadreminal cistern, while the smallest was near the brainstem and the anterior horn of the lateral ventricle. The most marked discrepancies were seen in the cranio-caudal (Y) axis of the hippocampus. Visual inspection of the contoured hippocampus revealed that the most important points localized in the hippocampus were lateral ventricles and location to the brainstem. The smallest deviations in hippocampal contours in all axes were observed near this location (Figure 6 and Figure 7).

## 4. Discussion

The development of modern RT techniques and their implementation in brain RT allows for more conformal planning. Delineation of structure such as the hippocampus in radiation oncology has become particularly important. Sparing the hippocampus during brain RT presents a considerable challenge for reasons such as its central location, unique anatomical shape as well as the fact that the hippocampus is not a separate structure of the brain well visualized by CT or MRI [22,23]. Additionally, differences in axial delineation of the hippocampus may be influenced by patient-specific variations in the shape of the organ caused by an age-related decline in hippocampal volume, neural infection or a psychiatric disease [24,25]. In our study, these case-specific hippocampal variations on CT or MRI images were the same for all doctors participating in the study.

The increase in the percentage of overlap between delineated contours of all cases reflected the participating physicians’ progress in the proper organ delineation process called “the learning curve”. The delineation of the hippocampus in this study was an individual learning process. The participants’ skills in contouring the hippocampus improved with each subsequent case. The contouring of 20 hippocampi by each doctor provided the physicians with a great opportunity of learning how to locate and delineate the structure properly. Clearly, the last contoured cases showed the smallest degree of variation in hippocampal volumes. Marginally larger volumes of LH in comparison to RH obtained in our study are similar to data obtained from studies based on automated segmentation of the hippocampus [26,27]. The hippocampal outlines in the first three cases were characterized by a larger volume than in the other seven cases, which may be associated with the participants’ uncertainty. Interestingly, contour delineation of LH was characterized by less variability in comparison to RH, which might be associated with the fact that 8 out of 10 participating doctors were right-handed. It should be assessed further in a larger study.

Nonetheless, the shape of the hippocampi contoured on CT scans fused with MRI was closer to the anatomical shape of the structure, particularly near the lateral ventricle and brainstem (Figure 6). The sizes and volumes of the hippocampi obtained in our study were similar to those reported in the literature [10,27,28] (length 4.5 cm, average width of body approximately 1 cm, mean hippocampal volume (delineated as dentate gyrus) for both LH and RH was 2.01 +/− 0.5 cm^3^). A similar inter-observer study that analyzed variation in hippocampus delineation for PCI demonstrated that the greatest degree of delineation inaccuracy occurred in the posterior and anterior-medial borders (SD range 1–2.5 mm) on computed local shape variation [10]. Similarly, in our study, deviation on the Z axis of the hippocampi ranged from 1.66 to 1.69 mm (Figure 6.)

The Hausdorf distance helped to estimate for comparison of the discrepancies in the three-dimensional structure of the hippocampus [23,24]. The results of the present study are similar to those reported by other authors (average HD—4.45 cm and 4.51 cm, respectively) [29,30]. In a similar study, analyzing difference between manual delineation of hippocampi on T1-weight MRI scans, the Dice similarity coefficient is in range between 0.18, 0.24 and 0.31 for the three cohorts [30]. In other studies using automatic contouring of hippocampi, the Dice coefficient index was higher compared to manually delineation hippocampi and was in range 0.65 to 0.95 [20,31]. The manually delineated hippocampi based on a deep learning course showed satisfying results, which could have a positive impact on improving delineation structures under RT planning.

Structural gadolinium-enhanced T1-weighted MRI provides the best contrast for delineation of the hippocampus from the surrounding structures [19]. Novel approaches, such as automated segmentation techniques, which utilize T1-weighted structural MR imaging for automatic segmentation of the hippocampus such as multi-atlas segmentation with brain-COLOR protocol [32]. However, some individuals have a history of neurological or psychiatric diseases which can be manifested by a disruption of the internal hippocampal structure, representing neuronal cell loss that may be difficult to appreciate visually [33]. In such cases, an automated atlas may fail to delineate the hippocampal body. An automated segmentation protocol for delineation of the hippocampus may correlate well in cases without any history of hippocampal pathology [32].

Different tools of hippocampus contouring are reported in the literature [25,30,34]. Some tools are accurate and highly useful in clinical practice since they facilitate better visualization of the location of the hippocampal body. The ventricle, quadreminal cistern and the splenium are known as landmarks [35]. Our study revealed that the critical point in delineation of the hippocampus and differentiation of the structure from the surrounding organs is the area near the quadreminal cistern, where largest discrepancies in hippocampus contouring were observed.

Precise delineation of the borders of the hippocampus is the basis for measuring the volume of the organ and estimating its shape. Automated contouring of the hippocampus does not ensure full anatomical plausibility, but it is a faster method [30,36]. Manual delineation of the structure appears to be more precise compared to automated methods for hippocampus segmentation in MRI [37,38]. It should also be pointed out that most RT departments lack automated algorithms for hippocampus delineation.

On the other hand, manual delineation of the hippocampus is a considerable challenge because of the low contrast of this structure in relation to other areas in the brain. By way of illustration, brain structure is clearly visible on MRI as opposed to CT. Furthermore, gadolinium, an MRI contrast medium, provides better localization as before of the choroid plexus, one of the anatomic areas of the hippocampus. Therefore, use of both sequences, with and without MRI T1 contrast, may contribute to a more precise delineation of the hippocampus [30,35,39]. To date, the T1 MRI sequence without contrast enhancement is the most frequently used in clinical practice.

## 5. Conclusions

Delineation of the hippocampus is problematic, difficult, and non-uniform. Training in proper contouring of the hippocampus as part of quality assurance in RT treatment planning is necessary to achieve optimal results in hippocampal-sparing brain RT.

## Data Availability

The data presented in this study are contained within the article.
